# Molecular Diagnosis of Myoclonus Epilepsy Associated with Ragged-Red Fibers Syndrome in the Absence of Ragged Red Fibers

**DOI:** 10.3389/fneur.2017.00520

**Published:** 2017-09-29

**Authors:** Sun Yeong Park, Se Hoon Kim, Young-Mock Lee

**Affiliations:** ^1^Departments of Pediatrics, Yonsei University College of Medicine, Seoul, South Korea; ^2^Departments of Pathology, Yonsei University College of Medicine, Seoul, South Korea

**Keywords:** myoclonus epilepsy with ragged-red fibers, mitochondria, ragged-red fibers, muscle, pathology, molecular diagnosis

## Abstract

Myoclonus epilepsy with ragged-red fibers (MERRFs), an inherited mitochondrial disorder, has characteristic morphological changes of ragged-red fibers (RRFs) in muscle biopsy, in the absence of which mitochondrial etiology is usually not considered in patients with phenotypes suggestive of MERRF. In these circumstances, MERRF can only be diagnosed using genetic analyses. The symptoms, pathological findings, and imaging results being age dependent, we can construct a protocol based on these characteristics to understand the disease’s natural course and to manage patients more effectively. The absence of RRFs should not preclude a MERRF diagnosis.

## Introduction

Myoclonus epilepsy with ragged-red fibers (MERRF) is an inherited mitochondrial disorder characterized by myoclonus epilepsy, ataxia, generalized seizures, and myopathy (1). Its estimated prevalence is about 1/400,000 in Northern Europe, although its prevalence in Asia is not established (2). MERRF affects the nervous system and skeletal muscles and is a genetically heterogeneous disease. Most patients have mutations in the mitochondrial deoxyribonucleic acid (mtDNA) tRNA^Lys^ gene (3), with the A8344G mutation responsible for 80–90% of the cases (4, 5). Morphological changes have been observed in muscle biopsies from patients with MERRF, including a substantial proportion of ragged-red fibers (RRFs), which are muscle fibers showing deficient cytochrome c oxidase (COX) activity, as well as the presence of COX-deficient vessels that are strongly immunoreactive for succinate dehydrogenase (6). In the absence of RFFs, mitochondrial etiology is not usually considered in patients with phenotypes suggestive of MERRF (7). We describe the case of a young patient with MERRF who carried the A8344G mutation, although muscle morphology and histochemical findings were normal.

## Case

A boy who was 4 years and 11 months old was admitted to our hospital for repeated myoclonic seizure symptoms. He was born by Cesarean section at 40 weeks of gestational age and had a birth weight of 2,060 g. He was an only child to his parents, and was born with no specific family history. At 6 months of age, the patient was suspected of having hearing impairment, mild hypotonia, and delayed development. However, he was never examined by a medical professional. An atonic seizure first occurred at around 4 years and 8 months of age. The patient later developed myoclonic seizures.

Upon admission, the patient’s weight was 21 kg (85th percentile), his height was 111 cm (75th percentile), and his head circumference was 51 cm (50th percentile). Ataxia and poor muscle tone were observed. He also showed poor language development and inarticulate pronunciation of words, which suggested overall development delay.

Results of blood tests for complete blood cell count, routine biochemical analysis, and creatine kinase were within the normal range. However, the patient had lactic acidosis and increased lactate levels (2.3–4.8 mmol/L; normal value: below 2.1 mmol/L) in blood, as indicated by repeated testing. The plasma amino acid test had no specific findings, but lactic aciduria was observed in the urine organic acid test.

**Figure 1:**
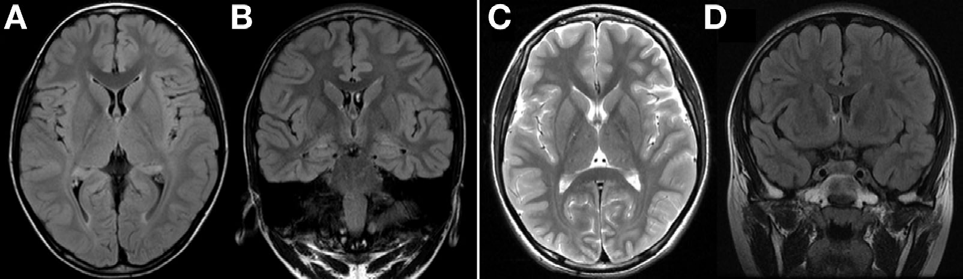
**(A,B)** Axial and coronal FLAIR MRI at 4 years of age showed normal findings. **(C,D)** Axial T2-weighted MRI and coronal FLAIR MRI at 6 years of age showed no significant interval change. FLAIR, fluid attenuated inversion recovery; MRI, magnetic resonance imaging.

Electroencephalography of the patient showed epileptiform discharges, such as generalized sharp and wave discharges, but there were no specific findings on magnetic resonance imaging (MRI) (Figure 1). There was no lactate peak on magnetic resonance spectroscopy. Cardiac function as assessed by echocardiography was normal. Myopia and astigmatism were found on ophthalmologic examination, but no abnormalities were found in the optic nerve or retina. Right sensory neural hearing loss was identified using pure tone audiometry.

**Figure 2:**
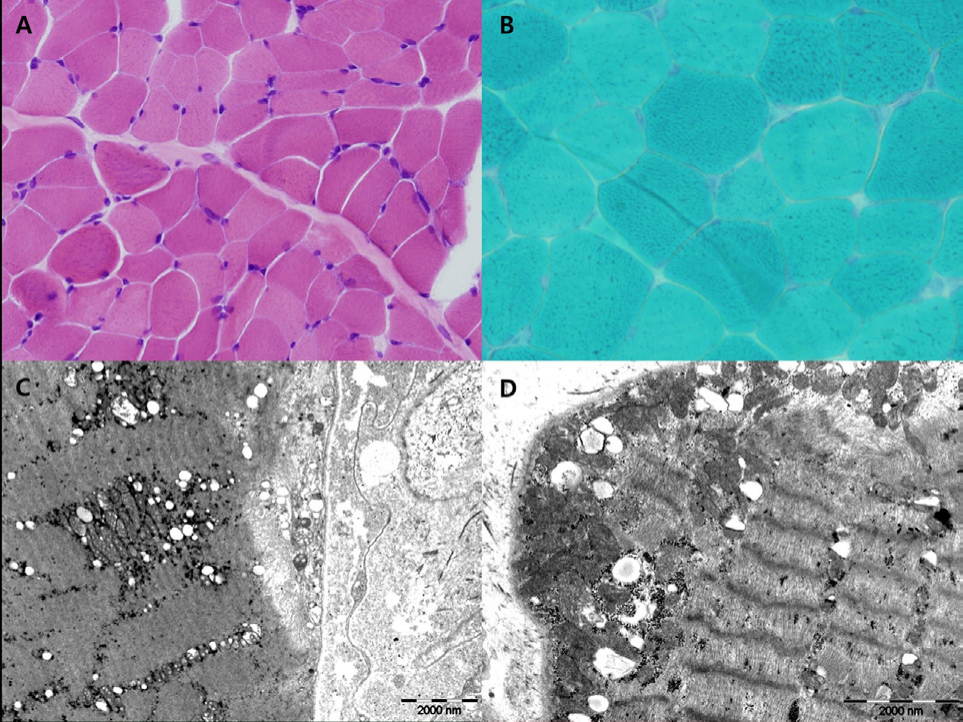
H&E **(A)** and Gomori-modified TRC **(B)** showed no ragged-red fiber (RRF). Ultrastructural study showed increased large mitochondria (megaconia) in the intermyofibrillar and subsarcolemmal area [**(C)** 10,000× and **(D)** 15,000×].

Myoclonus epilepsy with ragged-red fiber syndrome was suspected based on the presence of characteristic clinical symptoms with repeated lactic acidosis. Specific tests were conducted to confirm mitochondrial etiology. Muscle biopsies were performed surgically from the quadriceps muscle and routine histological, immunohistochemical, and electron microscopic (EM) examinations were conducted. Light microscopic examination of muscle tissues revealed no RRFs. No abnormal findings were revealed using the modified Gomori trichrome or other special immunohistochemical staining procedures. Although increased numbers of mitochondria were noted in the myofilament and EM examination revealed megaconia, the characteristic findings of mitochondrial myopathy were not observed (Figure 2). Biochemical assays to evaluate mitochondrial respiratory chain (MRC) enzyme activity were also performed. We observed decreased MRC complex I activity (below 10% of the reference range). Molecular genetic testing revealed the presence of the mitochondrial DNA A8344G mutation. The mutation burden was over 90%. Specimens were prepared from the patient’s muscle, fibroblast, and blood. These specimens had burdens of 97, 95, and 90%, respectively (Table 1). In addition, mitochondrial DNA from the patient’s mother indicated the presence of a 75% mutation burden in her blood. The patient had the clinical features of MERRF and the A8344G mutation, although no RRFs were observed using light microscopy (LM). The patient was finally diagnosed with mitochondrial disease.

**Table 1:** The mutant burden of mtDNA A8344G (%) in molecular genetic analysis.

	Skeletal muscle (%)	Skin fibroblast (%)	Blood (%)
Patient	97	95	90
Patient’s mother	Not available	Not available	75

*mtDNA, mitochondrial deoxyribonucleic acid*.

## Discussion

The classical MERRF diagnostic criteria, described by Fukuhara et al. in 1980, included the following typical manifestations of the disease: myoclonus, generalized epilepsy, cerebellar ataxia, and RRFs detected using muscle biopsy (8). With recent developments in genetic testing, MERRF can be diagnosed on the basis of specific gene mutations even if RRFs are absent (5). In this case, we suspected MERRF based on clinical manifestations. However, since the typical RRFs were absent on biopsy, MERRF was only diagnosed using genetic analyses. The occasional absence of RRFs in patients with MERRF due to the A8344G mutation has been reported previously (9). In fact, Gignoux et al. have reported that RRFs and COX-negative fibers may emerge later in the course of the disease (9). In other words, RRFs may not be detected when the child is younger and may appear at later follow-ups. In this case, the LM and immunohistochemical staining findings were normal, while EM revealed increased numbers of megaconia. Although this is not a characteristic finding of MERRF, it may suggest the presence of mitochondrial disease. Increased numbers of megaconia as detected using EM may be a significant finding in younger patients who do not exhibit RRFs in LM examinations. However, further studies are required to confirm this idea.

There is no positive correlation between the severity of clinical findings and the mutated mtDNA burden (3). However, the results of one study suggest that the threshold for expression of COX-negative fibers is a 90% burden of mutated mtDNA in individuals with A8344G mutations (10). In this case, the mutation burden in the patient’s blood specimen was 90% and that of his mother was 75%. Based on this observation, we can assume that the threshold for the expression of the A8344G gene mutation is between 75 and 90%. In our patient, high proportions of mtDNA carrying the A8344G mutation were found in the skeletal muscle, in fibroblasts, and in blood. The mutation burdens of the various specimens were different, a finding that is consistent with the important concepts of heteroplasmy and tissue specificity in mitochondrial disease.

No reports specifying the age of symptom onset for MERRF have been published. Although the main manifestation in patients with mitochondrial disease is the presence of myoclonus and epilepsy, other multisystemic alterations, such as deafness, exercise intolerance, dementia, peripheral neuropathy, short stature, abnormal cardiac conduction, cardiomyopathies, ophthalmoparesis, pigmentary retinopathy, and lipoma have been reported (4, 6). Given the presence of these heterogeneous clinical features, we can generally suspect MERRF based on the presence of global developmental delay and myoclonic seizures. In cases of mitochondrial disease, integrated approaches for assessment and treatment, such as growth status examination, audiologic evaluation, ophthalmologic evaluation, cardiac evaluation, assessment of cognitive ability, and physical therapy, are necessary. Such detailed assessments in patients with MERRF would help us to understand the natural course of the disease and to manage the patients with more care.

In this case, MRI findings at 4 years of age were normal. MRI performed 2 years later also revealed no specific findings. Conventional imaging studies of the brain, such as computed tomography or MRI, have confirmed that the gray matter is altered at an early stage in patients with MERRF, while changes in the white matter are more often observed in the later stages of the disease, and are never an isolated finding (11, 12). Cerebral, cerebellar, and brainstem atrophy occur as a result of progressive neuronal loss (4, 13, 14). Based on the patient’s MRI results, we can thus conclude that MRI does not provide definitive information for the diagnosis of MERRF. However, since MRI results can change with age, further MRI assessments during the course of the disease are essential.

## Conclusion

This study serves to illustrate the importance of molecular genetic analysis for the diagnosis of mitochondrial disease, especially in the absence of RRFs on muscle biopsy. Since the symptoms, pathological findings, and results of imaging studies are age-dependent, we can construct a protocol based on these characteristics to understand the disease’s natural course and to manage the patients more effectively. A diagnosis of MERRF should not be excluded even in cases without RRFs.

## Ethics Statement

This study was approved by the Institutional Review Board at Gangnam Severance Hospital. Written informed consent was obtained from the participant for the publication of this case report.

## Author Contributions

SP, SK, and YL designed the study protocol. SP and SK collected data and wrote the first draft of the manuscript under the mentorship of YL. All coauthors critically reviewed the manuscript.

## Conflict of Interest Statement

The authors declared no potential conflicts of interest with respect to the research, authorship, and/or publication of this article. The reviewer KS and handling Editor declared their shared affiliation.

## Abbreviations

MERRF, myoclonus epilepsy with ragged-red fibers; RRFs, ragged-red fibers; mtDNA, mitochondrial deoxyribonucleic acid; COX, cytochrome c oxidase; MRI, magnetic resonance imaging; EM, electron microscopy; MRC, mitochondrial respiratory chain; LM, light microscopy.
